# Efficacy of Wnt-1 monoclonal antibody in sarcoma cells

**DOI:** 10.1186/1471-2407-5-53

**Published:** 2005-05-24

**Authors:** Iwao Mikami, Liang You, Biao He, Zhidong Xu, Sonny Batra, Amie Y Lee, Julien Mazieres, Noemi Reguart, Kazutsugu Uematsu, Kiyoshi Koizumi, David M Jablons

**Affiliations:** 1 Thoracic Oncology Laboratory, Department of Surgery, Comprehensive Cancer Center, University of California, San Francisco, CA 94115, USA; 2 Department of Surgery II, Nippon Medical School, Tokyo 113-8602, Japan

## Abstract

**Background:**

Sarcomas are one of the most refractory diseases among malignant tumors. More effective therapies based on an increased understanding of the molecular biology of sarcomas are needed as current forms of therapy remain inadequate. Recently, it has been reported that Wnt-1/β-catenin signaling inhibits apoptosis in several cancers. In this study, we investigated the efficacy of a monoclonal anti-Wnt-1 antibody in sarcoma cells.

**Methods:**

We treated cell lines A-204, SJSA-1, and fresh primary cultures of lung metastasis of sarcoma with a monoclonal anti-Wnt-1 antibody. Wnt-1 siRNA treatment was carried out in A-204. We assessed cell death using Crystal Violet staining. Apoptosis induction was estimated by flow cytometry analysis (Annexin V and PI staining). Cell signaling changes were determined by western blotting analysis.

**Results:**

We detected Wnt-1 expression in all tissue samples and cell lines. Significant apoptosis induction was found in monoclonal anti-Wnt-1 antibody treated cells compared to control monoclonal antibody treated cells (p < 0.02). Similarly, we observed increased apoptosis in Wnt-1 siRNA treated cells. Blockade of Wnt-1 signaling in both experiments was confirmed by analyzing intracellular levels of Dishevelled-3 and of cytosolic β-catenin. Furthermore, the monoclonal anti-Wnt-1 antibody also induced cell death in fresh primary cultures of metastatic sarcoma in which Wnt-1 signaling was active.

**Conclusion:**

Our results indicate that Wnt-1 blockade by either monoclonal antibody or siRNA induces cell death in sarcoma cells. These data suggest that Wnt-1 may be a novel therapeutic target for the treatment of a subset of sarcoma cells in which Wnt-1/β-catenin signaling is active.

## Background

Sarcomas are highly malignant neoplasms that arise from mesenchymal tissues and the mechanism by which mesenchymal tissues undergo neoplastic transformation is largely unknown. Despite progress in the multidisciplinary treatment (surgery, chemotherapy, and radiation) of sarcomas, the results of these treatments in advanced disease remain unsatisfactory and the majority of these patients die from disseminated metastatic disease. Approximately 11,000 cases are diagnosed in the United States annually and 45 % of these patients will go on to die of their disease [1]. New therapies based on an improved molecular understanding of sarcomas are needed.

Recently, it has been reported that the Wnt pathway may be activated in several sarcomas [2-5]. Wnt signaling is essential for development and organogenesis [6,7]. It has been shown that the Wnt pathway is associated with tumor development and/or progression. We previously identified the overexpression of Dishevelled-3 (Dvl-3), a critical mediator of Wnt signaling, in non-small cell lung cancer and malignant pleural mesothelioma [8,9]. Wnt proteins, including Wnt-1, have been shown to be expressed in several cancers. We have developed a monoclonal anti-Wnt-1 antibody. In previous studies, we have demonstrated its efficacy in induction of apoptosis in human cancer cell lines and shown that the antibody lacks general toxicity in cells lacking the Wnt-1 protein [10-12]. This report suggests that the monoclonal anti-Wnt-1 antibody may also be efficacious in refractory sarcomas if Wnt-1/β-catenin signaling exists in these sarcomas. In the present study, we address this hypothesis and demonstrate a possible therapeutic role of this monoclonal anti-Wnt-1 antibody in the treatment of sarcoma cells.

## Methods

### Cell lines and tissue samples

Human sarcoma cell lines, A-204 (origin: muscle) and SJSA-1 (origin: bone) were obtained from the American Type Culture Collection (ATCC, Manassas, VA). A-204 and SJSA-1 were cultured in McCoy's 5a medium and RPMI 1640 respectively, with 10% fetal bovine serum, penicillin (100 IU/ml), and streptomycin (100 μg/ml). Both cells were cultured at 37°C in a humid incubator with 5% CO_2_. Fresh tissue samples of lung metastasis of sarcoma were obtained with consent from patients undergoing resection. They were cut into small pieces (1–2 mm in diameter), and digested with Collagenase A (Roche Applied Science, Indianapolis, Indiana) at room temperature for 2 hours according to manufacture's protocol. Single cells from the digestion were spun down and the cell pellets were washed twice using RPMI 1640 supplemented with 10% fetal bovine serum, penicillin (100 IU/ml) and streptomycin (100 μg/ml). Then, the cells were resuspended in the same medium and cultured in 6-well plates at 37°C in a humid incubator with 5% CO_2 _until they were ready for treatments. Other fresh tissue samples of lung metastasis of sarcoma were immediately snap-frozen in liquid nitrogen. They were kept at -170°C in a liquid nitrogen freezer before use.

### Western blotting

Whole cell lysates in tissue samples were obtained with T-Per Mammalian Protein Extraction Reagent (Pierce, Rockford, IL). Whole cell lysates in A-204 and SJSA-1 cell lines were obtained with M-Per Mammalian Protein Extraction Reagent (Pierce, Rockford, IL). Cytosolic proteins were prepared according to a previously described protocol [9]. The aliquots were separated on 4–15% gradient SDS-polyacrylamide gels and transferred to Immobilion-P (Millipore, Bedford, MA) membranes. Antigen-antibody complexes were detected by ECL blotting analysis system (Amersham Pharmacia Biotech, Piscataway, NJ). The following primary antibodies were used: Wnt-1 (custom-made, Rockland Inc., Gilbertsville, PA); Dvl-1, Dvl-2, Dvl-3, Survivin (Santa Cruz Biotechnology, Santa Cruz, CA); β-catenin (Transduction Laboratories, Lexington, KY); and β-Actin (Sigma Chemical Co., St Louis, MO).

### Antibody incubation with cells

The anti-Wnt-1 mouse monoclonal antibody (IgG1) was custom-made at Rockland Inc. (Gilbertsville, PA). The control monoclonal antibody (Sigma-Aldrich Co., St Louis, MO) used was the same subtype as the anti-Wnt-1 monoclonal antibody (IgG1). Cells were plated into six-well plates one day before experiments. Then normal medium was replaced by media containing antibodies at 10 μg/ml concentration and the cells were incubated at 37°C in a humid incubator with 5% CO_2_. Each experiment was performed at least 3 times.

### Apoptosis analysis

Cells were harvested by trypsinization and stained using an Annexin V-FITC Apoptosis kit (BioSource, Camarillo, CA), according to the manufacturer's protocol. Stained cells were immediately analyzed by flow cytometry (FACScan; Becton Dickinson, Franklin Lake, NJ). Early apoptotic cells with exposed phosphatidylserine but intact cell membranes bound to Annexin V-FITC but excluded propidium iodide (PI). Cells in necrotic or late apoptotic stages were labeled with both Annexin V-FITV and PI. Experiments were performed in triplicate and a total of 20000 cells were analyzed in each individual experiment.

### RNA interference

Cells were plated into a six-well plate with fresh media without antibiotics 24 hours before testing. The ion exchange high-performance liquid chromatography-purified siRNAs (Wnt-1 siRNA and nonsilencing siRNA control, >97% pure) were purchased from Qiagen-Xeragon (Germantown, MD). The siRNA-targeted human Wnt-1 is derived from a mRNA sequence (GGTTCCATCGAATCCTGCA) of human Wnt-1. The control (nonsilencing) siRNA does not target any known mammalian gene (the targeted sequence is AATTCTCCGAACGTGTCACGT). The lyophilized siRNA were dissolved in annealing buffer and reheated to 95°C for 1 minute followed by 1 hour at 37°C incubation. We followed the protocol described by Elbashir et al with some modifications [13]. After siRNA transfection, we incubated plates at 37°C before further analysis.

### Statistical analysis

Data shown represent mean values (± S.D.). Student's t-test was used for comparing different treatments and cell line.

## Results

### Detection of Wnt-1/ β-catenin signaling in tissue samples of metastatic sarcoma and sarcoma cell lines

**Figure 1:**
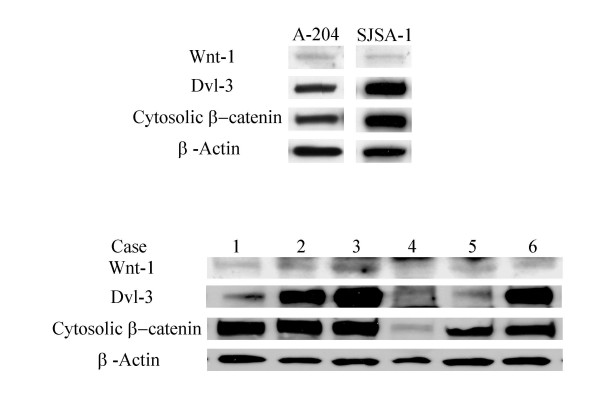
Tissue samples of metastatic sarcoma and sarcoma cell lines express Wnt-1 signaling mediators. Cases 1–6: tissue samples (1,3 and 6;osteosarcoma, 2;synovical sarcoma, 4;liposarcoma, 5;leiomyosarcoma).

The expression of Wnt-1, Dvl-1, 2, 3 and cytosolic β-catenin was analyzed in 6 tissue samples of metastatic sarcoma, A-204 and SJSA-1 cell lines. The expression of Wnt-1, Dvl-3 and cytosolic β-catenin was found in all tissue samples and both cell lines (Figure 1). Dvl-1 and Dvl-2 were minimally expressed or not expressed in all tissue samples (data not shown). These results indicate that Wnt-1/β-catenin signaling may be activated in sarcoma cells.

### The monoclonal anti-Wnt-1 antibody induces apoptosis in A-204 cell line

**Figure 2:**
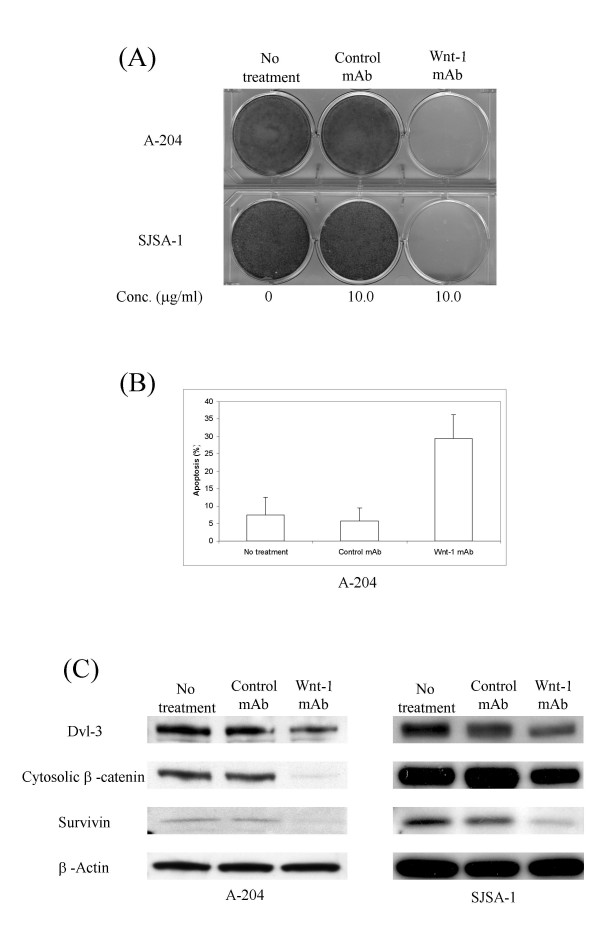
(A) 0.5% Crystal Violet staining of A-204 and SJSA-1 cell lines after the monoclonal anti-Wnt-1 antibody treatment (7 days after treatment). The concentration of both control and monoclonal anti-Wnt-1 antibodies used was 10 μg/ml. The monoclonal anti-Wnt-1 antibody induced cell death. (B) Apoptosis analysis using flow cytometry after the monoclonal anti-Wnt-1 antibody treatment (10 μg/ml for 6 days) in A-204 cell line. The results (percentage of apoptotic cells) are the mean ± SD (error bars). Significant apoptosis induction was observed in the monoclonal anti-Wnt-1 antibody treatment compared to the control monoclonal antibody treatment (p < 0.02). (C) Western analysis after the monoclonal anti-Wnt-1 antibody treatment (10 μg/ml for 6 days) in A-204 and SJSA-1 cell lines. β-Actin served as a loading control. Decreased levels of Dvl-3 and of cytosolic β-catenin confirmed the blockade of Wnt-1 signaling.

Next, the monoclonal anti-Wnt-1 antibody [10,11] was utilized for treating the sarcoma cell lines A-204 and SJSA-1. Cell death was found after 7 days of treatment with the monoclonal anti-Wnt-1 antibody (Figure 2A). By flow cytometry analysis, we found significant apoptosis induction in A-204 cell line (Figure 2B)(p < 0.02). In contrast, no noticeable effect was found after control monoclonal antibody treatment.

### The monoclonal anti-Wnt-1 antibody inhibits Wnt-1/β-catenin signaling

The blockade of Wnt-1 signaling in A-204 and SJSA-1 cell lines was confirmed by analyzing the levels of Wnt-1 downstream mediators (Figure 2C). Dvl-3 and β-catenin protein levels decreased after the monoclonal anti Wnt-1 antibody treatment. Survivin, an inhibitor of apoptosis, was also downregulated.

### RNA interference

**Figure 3:**
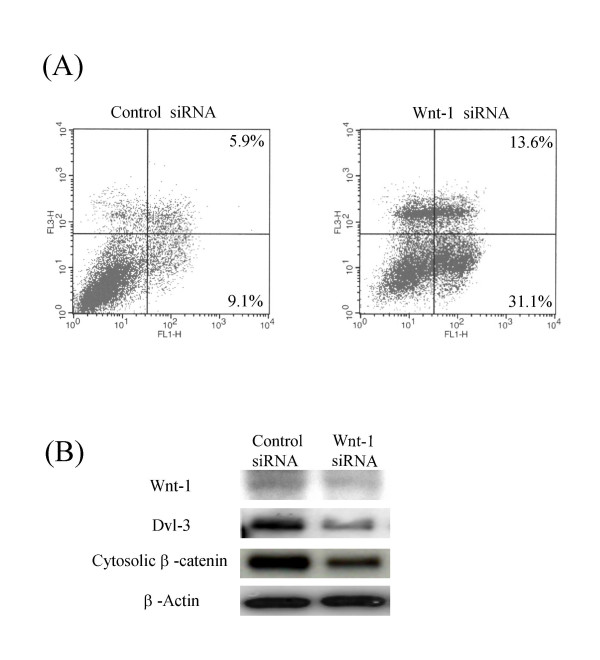
(A) Apoptosis and (B) expression of Wnt-1 signaling mediators analysis after Wnt-1 siRNA treatment (200 nM for 4 days) in A-204 cell line. The results were similar to that of the monoclonal anti-Wnt-1 antibody treatment.

Next, we investigated the effects of silencing Wnt-1 expression through RNA interference. Apoptosis induction by Wnt-1 siRNA was similar to that by anti-Wnt-1 monoclonal antibody (Figure 3A). Moreover, we confirmed the blockade of Wnt-1 signaling in A-204 cells after Wnt-1 siRNA treatment (nonsilencing siRNA as control) by western blot analysis (Figure 3B).

### The monoclonal anti-Wnt-1 antibody induces cell death in fresh primary cultures of metastatic sarcoma

**Figure 4:**
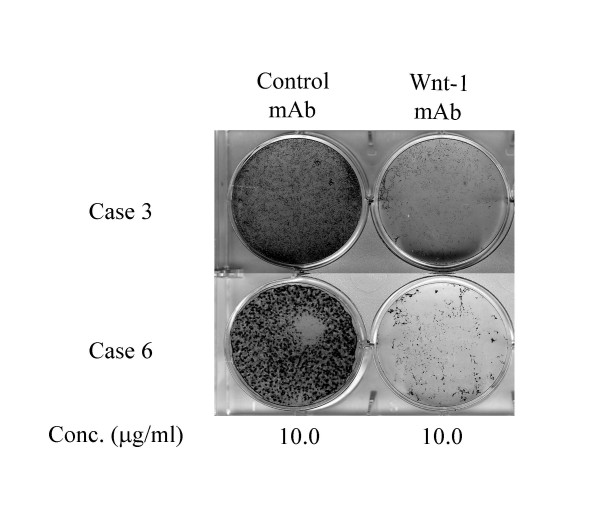
0.5% Crystal Violet staining of fresh primary cultures of metastatic osteosarcoma after the monoclonal anti-Wnt-1 antibody treatment. The concentration of both control and monoclonal anti-Wnt-1 antibodies used was 10 μg/ml. The monoclonal anti-Wnt-1 antibody also induced cell death in these fresh samples.

After confirmation of the efficacy of monoclonal anti-Wnt-1 antibody treatment in A-204 and SJSA-1 cells, we tested its ability to induce cell death in fresh primary cultures of metastatic osteosarcoma that has activated Wnt-1/β-catenin signaling (Cases 3 and 6). The cell death was found after 5 to 7 days of the antibody treatment, whereas no noticeable effect was found in these fresh samples after control monoclonal antibody treatment (Figure 4).

## Discussion

Wnt signaling consists of an intracellular cascade that involves Frizzled, Dvl, glycogen synthase kinase-3β, β-catenin and T Cell Factor (TCF)/Lymphocyte Enhancer Binding Factor (LEF) [14,15]. It is thought that a component of cancer induction and progression results from elevated intracellular β-catenin levels [16]. It has been reported that Wnt signaling is associated with the progression of osteosarcoma [17]. Translocation of β-catenin to the cytoplasm and/or nucleus of osteosarcoma cells was detected in five resected cases of pulmonary metastasis, although seven primary osteosarcoma cells that did not metastasize for more than five years did not show β-catenin expression [18].

Wnt-1 was originally described as a proto-oncogene in mouse mammary tumor induced by mouse mammary tumor virus [19]. Chen et al. reported that Wnt-1 signaling inhibited apoptosis through β-catenin and that cells expressing Wnt-1 resisted apoptosis induction by chemotherapy [20]. Recently, it has been reported that a polyclonal anti-Wnt-1 antibody can downregulate β-catenin/TCF activity and induce apoptosis in head and neck squamous cell carcinoma [21]. In addition, we demonstrated that both a monoclonal anti-Wnt-1 antibody and Wnt-1 siRNA induced apoptosis in human cancer cells [10-12]. These reports strongly suggest that Wnt-1 signaling plays a critical role in the survival of malignant cells. We treated A-204, SJSA-1 cells and fresh primary cultures with the monoclonal anti-Wnt-1 antibody after confirmation of Wnt-1/β-catenin signaling in sarcoma cells. The antibody induced cell death in these sarcomas and decreased levels of Dvl-3 and β-catenin in A-204 and SJSA-1 cell lines. Furthermore, Wnt-1 siRNA induced similar effects. These results indicate that Wnt-1 signaling may play a critical role in the survival of a subset of sarcoma cells. However, much work remains in order to gain a clear understanding of the pathway.

## Conclusion

Our results indicate that the monoclonal anti-Wnt-1 antibody induces cell death in sarcoma cells. These findings suggest that Wnt-1 inhibition may be of therapeutic interest in a subset of sarcomas in which Wnt-1/β-catenin signaling is active.

## Competing interests

We have not received reimbursements, fees, funding, or salary for our work. We have licensed our Wnt antibody to a start-up company commercializing Wnt based therapeutics (David M. Jablons, Liang You, Zhidong Xu, and Biao He). Our monoclonal Wnt-1 antibody is pending patent.

## Authors' contributions

IM carried out western blotting, cell staining, RNA interference and apoptosis analysis. LY, BH, ZX, KU, KK and DJ conceived of the study, and participated in its design and coordination. SB, AY, JM and NR drafted the manuscript.

All authors read and approved the final manuscript.

## Pre-publication history

The pre-publication history for this paper can be accessed here:
